# Multiple Primary Cancers as an Independent Criterion for Germline Testing: Comparison with Guideline-Based Criteria

**DOI:** 10.3390/jcm14207310

**Published:** 2025-10-16

**Authors:** Kabsoo Shin, Hoon Seok Kim, Hee Yeon Lee, Jong Min Baek, MyungAh Lee, Sook Hee Hong, Jieun Lee, Se Jun Park, Myungshin Kim, In Sook Woo

**Affiliations:** 1 Division of Medical Oncology, Department of Internal Medicine, Seoul St. Mary’s Hospital, College of Medicine, The Catholic University of Korea, Seoul 06591, Republic of Korea; kabsoo.shin@catholic.ac.kr (K.S.);; 2 Cancer Research Institute, College of Medicine, The Catholic University of Korea, Seoul 06591, Republic of Korea; 3 Department of Laboratory Medicine, Seoul St. Mary’s Hospital, College of Medicine, The Catholic University of Korea, Seoul 06591, Republic of Korea; 4 Catholic Genetic Laboratory Center, Seoul St. Mary’s Hospital, College of Medicine, The Catholic University of Korea, Seoul 06591, Republic of Korea; 5 Division of Medical Oncology, Department of Internal Medicine, Yeouido St. Mary’s Hospital, College of Medicine, The Catholic University of Korea, Seoul 07345, Republic of Korea; urloved@catholic.ac.kr; 6 Department of Surgery, Yeouido St. Mary’s Hospital, College of Medicine, The Catholic University of Korea, Seoul 06591, Republic of Korea; jmbaek@catholic.ac.kr

**Keywords:** multiple primary cancers, germline genetic testing, cancer predisposition, hereditary cancer syndromes, pathogenic variants

## Abstract

**Background**: Multiple primary cancers (MPCs) often indicate an underlying hereditary predisposition. Current genetic testing guidelines mainly target specific cancer types, potentially missing MPC patients who do not meet these criteria. This study evaluated the utility of MPCs as an independent criterion for germline genetic testing by comparing the pathogenic variant (PV) diagnostic yields of guideline-based and MPC-based testing. **Methods**: Between June 2022 and June 2023, we prospectively enrolled 62 patients diagnosed with two or more pathologically confirmed primary cancers. Patients were classified into a Guideline group (*n* = 29), which met NCCN/ACMG testing criteria, and a Non-Guideline group (*n* = 33) classified solely on the MPC status. Germline testing was performed using a 25-gene hereditary cancer panel and by BRCA1/2 next-generation sequencing. **Results**: Pathogenic variants were identified in four patients (6.5%): two in the Guideline group (*CHEK2*, *BRCA2*) and two in the Non-Guideline group (*ATM*, *TP53*). Diagnostic yields were similar in the two groups (6.9% vs. 6.1%, respectively, *p* = 0.763). Of eight patients with ≥3 primary cancers, one patient (12.5%) had a clinically significant *TP53* deletion without meeting Li-Fraumeni syndrome criteria. All PV-positive patients had a family history of cancer. Variants of uncertain significance were identified in 25 (40.3%) of the 62 study subjects. **Conclusions**: Germline genetic testing based solely on MPC had a diagnostic yield comparable to guideline-based testing. MPC could be considered as an independent criterion for genetic testing to improve the identification of a hereditary cancer predisposition.

## 1. Introduction

Multiple Primary Cancers (MPCs) are characterized by the occurrence of two or more distinct primary malignancies in the same individual over a lifetime [1]. The definitions of MPCs used vary slightly, but widely accepted criteria were provided by the Surveillance, Epidemiology, and End Results (SEER) program and the International Association of Cancer Registries/International Agency for Research on Cancer (IACR/IARC) [2]. The reported frequency of MPCs ranges from 5% to 17% in cancer populations [1,3], and this incidence is increasing due to improved cancer survival rates, advances in diagnostic techniques, improved screening programs, and greater clinical awareness [1,4].

MPC development is the result of complex interplay between genetic predisposition, prior cancer treatment, environmental exposures, and lifestyle-related factors [1]. Notably, MPCs often indicate underlying hereditary cancer predisposition syndromes, in which germline pathogenic variants (PVs) in specific genes significantly increase the lifetime risk of MPCs [5]. Identifying such genetic predispositions is critical for guiding treatment decisions, implementing risk-reduction interventions, tailoring cancer screening strategies, and conducting cascade testing for at-risk family members [6].

Current clinical guidelines recommend germline genetic testing primarily based on cancer-specific factors such as age at diagnosis, family history, tumor histology, and other clinical features [7,8,9]. However, these guidelines are continuously evolving as our understanding of cancer genetics expands. For instance, while testing was initially limited to *BRCA1/2* for breast and ovarian cancers, current recommendations have expanded this to include multi-gene panel testing for these and other cancer types [10,11].

Recently, there has been growing interest in universal approaches to germline genetic testing beyond the traditional guideline-based approach [12,13]. Comparative research studies on guideline-based testing and universal approaches have shown that the latter can identify pathogenic variants (PVs) missed using traditional criteria [3,10,12]. Several studies have demonstrated the utility of MPC-focused germline testing, regardless of family history, and reported substantial detection rates for germline PVs [14]. Furthermore, multi-gene panel assays have replaced single-gene testing and enable more comprehensive evaluations of genetic risk [11].

In Republic of Korea, multiple studies have explored the incidence, clinical significance, and genetic underpinnings of MPCs. Large-scale hospital-based studies have reported the incidence of MPCs in Korean cancer patients to be approximately 2.2–2.3%, noting that the presence of an additional primary cancer is associated with poorer survival outcomes [15,16]. These findings emphasize the clinical importance of recognizing MPC status in routine care. Furthermore, research focused on high-risk Korean cohorts has revealed a significant genetic contribution; pathogenic germline variants were identified in 40% of patients with MPCs involving hematologic malignancies (e.g., *ATM*, *TP53*) and in 36.8% of young patients with both gastric and colorectal cancers (Lynch syndrome-associated variants) [17,18]. Reinforcing this link, another study found that Korean cancer patients with germline *PALB2* variants were significantly more likely to develop MPCs compared to non-carriers (22.7% vs. 8.3%) [19]. These population- and hospital-based data from Republic of Korea provide crucial context, revealing a heterogeneous tumor spectrum and a significant genetic contribution to MPCs among Korean patients. While these studies highlight the genetic link, they do not directly address the clinical utility of using MPC status as a primary criterion for genetic testing compared to guideline-based criteria.

The results of many germline tests for MPC align with established guidelines for specific cancers, but there are instances where MPC patients do not meet current guideline-based criteria. These include patients with cancers that lack established genetic testing recommendations or age, family history, or histopathological characteristics that do not fulfill guideline thresholds. However, the comparative detection rates of cases meeting both guideline and MPC criteria versus those meeting only MPC criteria have not been established.

This study was undertaken to prospectively evaluate the clinical utility of germline testing based on meeting the single criterion of MPC by comparing the PV detection rates of these patients and patients eligible under current guidelines, and thus, to determine the value of MPC-based germline testing in clinical practice.

## 2. Materials and Methods

### 2.1. Patient Selection

Patients with two or more pathologically confirmed primary cancers were prospectively registered at Seoul St. Mary’s Hospital or Yeouido St. Mary’s Hospital from June 2022 to June 2023. Multiple primary cancers were defined according to criteria proposed by the International Agency for Research on Cancer (IACR). This definition considers tumors as separate primaries if they originate in different sites/organs or exhibit different histologies/morphologies when in the same site. Enrollment criteria were broad, and patients were enrolled regardless of family history, disease site, cancer stage, age, or sex. No exclusions were applied to maximize the generalizability of the findings. All participants provided written informed consent before inclusion in the study. The study was conducted following the principles outlined in the Declaration of Helsinki and was reviewed and approved by the Institutional Review Board of Seoul St. Mary’s Hospital and Yeouido St. Mary’s Hospital (IRB approval number: KC22OIDI0882, SC22TISI0081).

### 2.2. The Guideline and Non-Guideline Groups

The enrolled patients were divided into two groups based on their eligibility for germline genetic testing according to established clinical guidelines. The Guideline group included patients who met the current NCCN (National Comprehensive Cancer Network) and ACMG (American College of Medical Genetics and Genomics) criteria for germline genetic testing specific for their cancer types [7,8,9]. These criteria included tumor-specific factors such as age at diagnosis (<45–50 years for breast cancer, <60 years for colorectal cancer), tumor characteristics (triple-negative breast cancer, medullary thyroid cancer), and a family history of relevant cancers. The Non-Guideline group consisted of patients who did not meet these guideline-based criteria and therefore underwent genetic testing based on meeting the multiple primary cancer (MPC) criterion. The most recent ACMG and NCCN guidelines were applied at the time of analysis.

### 2.3. Germline Genetic Testing and Variant Interpretation

Genomic DNA was extracted from peripheral blood using the QIAsymphony DSP DNA Mini Kit (Qiagen, Hilden, Germany). NGS was conducted using a targeted capture sequencing panel (the Hereditary Cancer Research Panel, Thermo Fisher Scientific Inc., Waltham, MA, USA), which included 25 hereditary cancer-related genes involved in DNA repair and the mismatch repair pathway: *APC*, *ATM*, *BARD1*, *BMPR1A*, *BRIP1*, *CDH1*, *CDK4*, *CDKN2A*, *CHEK2*, *EPCAM*, *MLH1*, *MRE11A*, *MSH2*, *MSH6*, *MUTYH*, *NBN*, *PALB2*, *PMS2*, *PTEN*, *RAD50*, *RAD51C*, *RAD51D*, *SMAD4*, *STK11*, and *TP53* [20]. This panel was selected based on its comprehensive coverage of well-established cancer predisposition genes associated with various solid tumors. Additionally, we also conducted BRCA1/2 NGS assay using BRCAaccTest PLUS panel (NGeneBio, Seoul, Republic of Korea) which additionally covered a *BRCA1* promoter region [21]. The test methods and variant calling bioinformatic pipelines for single-nucleotide variants (SNVs), small insertions or deletions, and copy number variants (CNVs) were conducted as previously described [20,21]. Sanger sequencing or multiplex ligation-dependent probe amplification (MLPA) was conducted to confirm sequence variations or copy number changes. MLPA probe mixes P002 and P045 were used to screen large genomic rearrangements in *BRCA1* and *BRCA2*/*CHEK2*, respectively, while P087 and P077 were used for confirmation (MRC-Holland, Amsterdam, The Netherlands). Also, P003 was selected for *MLH1*/*MSH2*, P008 for *PMS2*, P056 for *TP53*, P072 for *MSH6*-*MUTYH*, and P225 for *PTEN*, according to the manufacturer’s instructions. The amplified products were analyzed by capillary electrophoresis on an ABI 3500 Genetic Analyzer (Applied Biosystems, Foster City, CA, USA). Data analysis was conducted with Coffalyser.Net software (v220513.1739; MRC Holland, Amsterdam, The Netherlands). Intensity ratios were interpreted as follows: 0.8–1.2 indicated a normal copy number (wild type) and 0.40–0.65 indicated a heterozygous deletion.

Identified variations were classified according to the American College of Medical Genetics and Genomics and the Association for Molecular Pathology guidelines and incorporated population data, predictive in silico analyses, functional studies, familial segregation, inheritance or de novo occurrence, allelic information, and data from established databases [22,23]. Variants were categorized as pathogenic, likely pathogenic, variant of uncertain significance (VUS), likely benign, or benign, following Human Genome Variation Society nomenclature standards [24]. Population allele frequencies were obtained from gnomAD v2.1.1. Full variant-level details for PV/LPV and VUS are provided in Supplementary Table S1.

### 2.4. Statistical Analysis

The Guideline and Non-Guideline groups were compared using statistical tests appropriate for data type. Categorical variables were analyzed using the chi-square test or Fisher’s exact test when expected cell counts were less than five. Continuous variables were evaluated with independent *t*-tests after confirming distribution normality. The primary outcome measure was the comparison of pathogenic variant (PV) detection rates between the two groups. Secondary analyses included PV detection rates stratified by number of primary cancers (2 vs. ≥3) and by presence of family history. Statistical significance was defined as a *p*-value < 0.05. All statistical analyses were performed using R Statistical Software (version 4.2.2; R Core Team, Vienna, Austria).

## 3. Results

### 3.1. Baseline Characteristics

**Figure 1:**
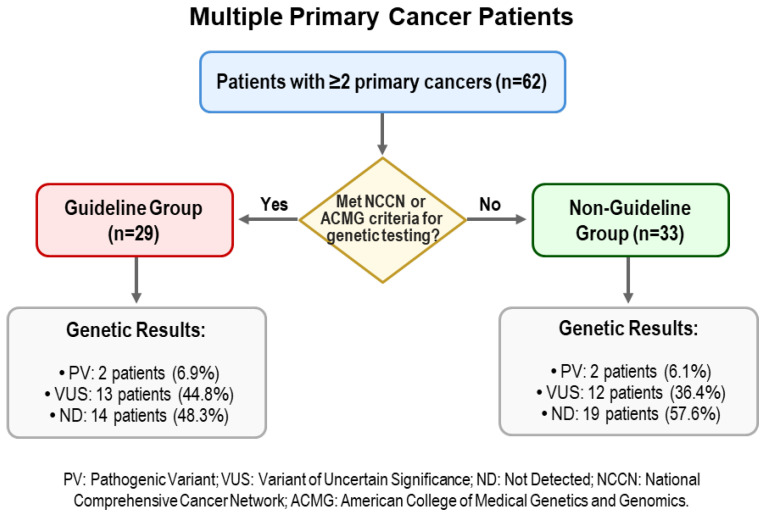
Flow chart of patient classification and pathogenic variant detection rates in multiple primary cancer patients.

A total of 62 patients with MPC were enrolled; 28 (45.1%) were male and 34 (54.8%) were female, and median age at testing was 66 years (range 39–84). The majority (*n* = 54, 87.1%) had two primary cancers, while seven (11.3%) had three, and one patient had four. Twenty-nine patients (46.8%) had a family history of cancer in a first-degree relative. Based on genetic testing eligibility, 29 patients (46.8%) met established NCCN/ACMG guidelines (the Guideline group), and 33 patients (53.2%) were eligible based on MPC status alone (the Non-Guideline group) (Figure 1).

Demographic and clinical characteristics were significantly different in the two groups. Median age was higher in the Non-Guideline group (70 vs. 63 years, *p* = 0.001), and this group also had an older age at diagnosis for the first (61 vs. 53 years, *p* = 0.002) and second cancers (67 vs. 59 years, *p* = 0.006). In addition, the proportion of females was significantly higher in the Guideline group (75.9% vs. 36.4%, *p* = 0.004). No significant intergroup difference was observed for number of primary cancers (two vs. ≥three cancers) or a family cancer history (Table 1).

### 3.2. Types of Cancers

**Figure 2:**
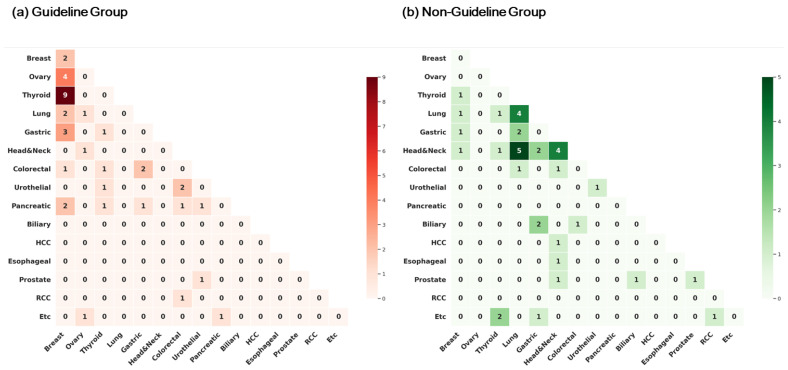
Pairwise distribution of tumor types in multiple primary cancer (MPC) patients, stratified by germline genetic testing criteria. (**a**) Guideline group (*n* = 29): Heatmap showing tumor pair frequencies in patients who met current NCCN/ACMG criteria. (**b**) Non-Guideline group (*n* = 33): Heatmap of tumor pair frequencies in patients who underwent testing based solely on the MPC criterion.

One hundred and thirty-two distinct primary cancers were identified in the 62 patients. The most common cancer types were breast cancer (22 cases, 16.5%), thyroid cancer (15 cases, 11.3%), lung cancer (14 cases, 10.5%), head and neck cancer (13 cases, 9.8%), gastric cancer (12 cases, 9.1%), and colorectal cancer (10 cases, 7.5%) (Table S2). Furthermore, distributions of cancer types differed in the two groups. In the Guideline group (*n* = 63 cancers), breast cancer was the most common (18 cases, 28.6%), followed by thyroid cancer (10 cases, 15.9%) and pancreatic cancer (7 cases, 11.1%), whereas in the Non-Guideline group (*n* = 69 cancers), lung cancer (12 cases, 17.4%) and head and neck cancer (12 cases, 17.4%) were the most common, followed by gastric cancer (7 cases, 10.1%). Figure 2 illustrates cancer types and tumor pair associations observed in the study groups.

### 3.3. Genetic Variants Identified

Of the 62 patients, one pathogenic or likely pathogenic variant (PV) was identified in four patients (6.5%). In addition, variants of uncertain significance (VUS) were identified in 25 patients (40.3%), while no variants were found in the remaining 33 patients (53.2%). The distribution of variants was similar in the two study groups. In the Guideline group, 2 patients (6.9%) had PVs (*CHEK2* and *BRCA2*), 13 patients (44.8%) had at least one VUS, and 14 patients (48.3%) had no detectable variant. In the Non-Guideline group, 2 patients (6.1%) had PVs (*ATM* and *TP53*), 12 patients (36.4%) had at least one VUS, and 19 patients (57.6%) had no detectable variant. The distributions of genetic findings in the two groups were not significantly different (χ^2^, *p* = 0.763). The specific details of the identified PVs are provided in Table 2, and additional information from published literature and ClinVar regarding their known cancer associations is summarized in Supplementary Table S3.

The four PVs identified in this study represent diverse molecular alterations and cancer associations. In the Guideline group, one patient had a *CHEK2* splice site variant (c.846 + 1G > T) and was diagnosed with breast and ovarian cancers at age 61. Another patient harbored a *BRCA2* frameshift variant (c.5576_5579del, p.(Ile1859Lysfs)) and was diagnosed with breast cancer at age 36 and pancreatic cancer at age 54. In the Non-Guideline group, one patient carried an *ATM* nonsense variant (c.103C > T, p.(Arg35Ter)) and was diagnosed with tongue cancer at age 59 and breast cancer at age 79. The fourth patient had a *TP53* large deletion involving the entire gene (17p13.1del) and presented with four primary cancers: stomach cancer at age 53, nasal cavity cancer at age 54, non-small cell lung cancer at age 60, and tongue cancer at age 64. Notably, this patient with four primary cancers would not have met traditional criteria for Li-Fraumeni syndrome genetic testing despite harboring a germline *TP53* deletion. All patients with a PV reported a family history of cancer, although the specific cancers in relatives were not always characteristic of the syndrome associated with the identified gene variant (Table 2).

When the analysis was limited to patients with a family history of cancer (*n* = 29), the PV detection rate was 13.8% overall, and rates in the Guideline group (13.3%) and Non-Guideline group (14.3%) were comparable (χ^2^, *p* = 0.544). Further analysis by number of primary cancers revealed that among patients diagnosed with two primary cancers (*n* = 54), PVs were detected in 3 patients (5.6%); 2 of 24 in the Guideline group (8.3%) and 1 of 30 in the Non-Guideline group (3.3%). Among patients with three or more primary cancers (*n* = 8), 1 patient (12.5%) harbored a PV. Notably, no PV was detected in the 5 patients in the Guideline group with ≥3 cancers, while 1 of 3 patients (33.3%) in the Non-Guideline group with ≥3 cancers had a PV.

Thirty-four VUSs were identified in 16 genes. The most frequently affected genes were *ATM* (5 VUS), *CHEK2* (4 VUS), *MUTYH* (4 VUS), *APC* (3 VUS), *MSH2* (3 VUS), and *NBN* (3 VUS). The prevalence of a VUS was slightly but non-significantly higher in the Guideline group (44.8%) than in the Non-Guideline group (36.4%) (*p* = 0.498). Detailed variant-level annotations, including HGVS nomenclature, ClinVar accessions/classifications, and population allele frequencies, are provided in Supplementary Table S1.

## 4. Discussion

In this study, we prospectively evaluated the clinical utility of germline genetic testing based solely on the criterion of multiple primary cancers (MPCs) and compared pathogenic variant (PV) detection rates with those obtained through traditional guideline-based criteria. Our findings suggest that MPC alone, that is, independently of existing cancer-specific guidelines, provides a clinically meaningful indication for germline genetic testing.

The overall detection rate of PVs in our MPC cohort was 6.5%. This rate is somewhat lower than the range of 8–30% reported in other various hereditary cancer studies [1,4,5,14]. Furthermore, it is notably more modest when compared to reports from specific high-risk Korean MPC cohorts, such as a case series involving hematologic malignancies (40% detection rate) or a study on young-onset gastric and colorectal cancer patients (36.8% detection rate) [17,18]. This discrepancy likely reflects our study’s inclusive and prospective enrollment criteria, which recruited patients with any two primary cancers regardless of age, cancer type, or specific high-risk features, in contrast to the aforementioned studies that focused on highly selected patient groups. Additionally, the small sample size may partially account for the modest detection rate, underscoring the need for further studies involving larger and more diverse populations.

More than half of the MPC patients (53.2%) enrolled did not meet existing guideline criteria for germline testing based on individual cancer types. This finding aligns with previous studies reporting that 55.5% and 48.4% of patients with pathogenic variants, respectively, would not have been identified by guideline-based testing alone [3,10]. Patients not meeting existing guidelines in our study mainly fell into three categories: (1) patients diagnosed with a common cancer such as lung cancer, typically less associated with germline variants; (2) cancers primarily attributed to environmental factors (e.g., head and neck cancers); and (3) rare cancers (e.g., biliary tract cancers) without clear genetic testing recommendations. These results highlight a significant gap in current testing practices that may result in missing leave many MPC patients.

When we directly compared the Guideline group and the Non-Guideline group, PV detection rates were nearly identical (6.9% vs. 6.1%, respectively). This similarity is particularly significant as it challenges established reliance on cancer-specific genetic testing criteria alone, especially since clinically impactful PVs in genes like *ATM* and *TP53* were identified in the Non-Guideline group.

The clinical value of this MPC-based approach is clearly illustrated by the detection of a germline *TP53* deletion in a patient from this group. We acknowledge the valid point that this case is clinically atypical for Li-Fraumeni syndrome (LFS). The patient presented with four primary cancers (stomach, nasal cavity, lung, and tongue) and had a first-degree relative with lung cancer, yet none of these are core-spectrum tumors (e.g., sarcomas, early-onset breast cancer, brain tumors) that would typically trigger a suspicion of LFS [25]. This atypical presentation is precisely why the case is informative; it demonstrates a scenario where a high-penetrance pathogenic variant would almost certainly be missed by traditional, criteria-based genetic testing [26]. The identification of this *TP53* deletion was therefore possible only because the presence of MPCs itself was used as an independent indication for testing, highlighting the critical utility of our proposed approach in identifying at-risk individuals who fall outside of current guidelines.

This theme of atypical presentations was also observed in other carriers. For instance, the *ATM* PV-positive patient was diagnosed with breast cancer at an advanced age (79 years) and tongue cancer at 59 years, a tumor type less commonly associated with the *ATM*-related cancer spectrum. Although a viral etiology for the tongue cancer could not be definitively excluded due to unavailable historical pathology records from an external institution, the identification of the germline *ATM* pathogenic variant in this individual with two distinct primary cancers remains a critical and independent finding. This germline information has significant clinical implications for the patient’s lifetime cancer risk management and for the predictive testing of at-risk family members, irrespective of the specific etiology of any single tumor.

These cases collectively highlight the considerable heterogeneity in tumor spectra among carriers of pathogenic variants. However, it is important to note that given the absence of familial segregation analyses and the study’s design, we refrain from inferring penetrance or expressivity from our cohort.

In our subgroup analysis, patients with three or more primary cancers exhibited a PV detection rate of 12.5%, reinforcing the findings of Bychkovsky et al. (2022), who observed that patients with a higher number of primary cancers had increased prevalence of pathogenic variants (13.1% with 2 primary cancers, 15.9% with 3 primary cancers, and 18.0% with ≥4 primary cancers) [14]. Specifically, one of three Non-Guideline group patients with ≥3 primary cancers harbored a significant *TP53* deletion. This observation underscores the importance of evaluating germline genetic testing, especially in patients with multiple cancers, regardless of classical syndromic criteria.

Variants of uncertain significance (VUS) were identified in 40.3% of our patients, concurring with prior reports that highlighted the interpretative challenges faced in Asian populations due to underrepresentation in genomic databases, which predominantly reflect European ancestries [3,27,28]. In clinical practice, managing these findings requires a clear framework. Crucially, a VUS result is not considered clinically actionable for definitive medical interventions like risk-reducing surgery; instead, patient management should be guided by personal and family history. Key strategies include comprehensive genetic counseling, long-term follow-up for variant reclassification, and segregation analysis in family members where feasible. To address the root cause of this high VUS rate, increasing genomic data diversity and improving interpretation guidelines are urgently needed. Contributing data from diverse cohorts like ours is an essential part of this effort. This challenge underscores why an MPC-focused genetic testing strategy is a valuable complement to existing guidelines. Reliance solely on traditional NCCN or ACMG criteria may result in significant gaps in identifying hereditary cancer predispositions, especially within underrepresented ethnic populations such as ours. Consequently, developing more inclusive, ethnicity-specific testing criteria could significantly improve both variant classification and overall patient management outcomes.

Our findings raise the important question of how MPC status might be incorporated into future germline testing guidelines. Given the modest sample size and single-country cohort of this study, we do not propose MPC as a universal indication for germline testing at this stage. Instead, our data suggest that certain subgroups of MPC patients—even those not meeting traditional guideline criteria—may represent a high-yield population for whom germline testing could be particularly beneficial. Specifically, patients with three or more primary cancers demonstrated a detection rate of 12.5%, and those with two primary cancers plus a first-degree family history of cancer showed a comparable yield of 13.8%. These observations suggest the potential clinical utility of using MPC status as an independent testing criterion in such high-risk contexts. Further large, multi-ethnic studies are required to validate these specific yields and to determine if MPC could ultimately serve as a broader trigger for germline testing.

### Limitations

This study has several limitations. First, a primary limitation is the study’s relatively small sample size (*n* = 62). This impacts the statistical power of our analyses, meaning that the lack of a significant difference in PV detection rates between the Guideline and Non-Guideline groups should be interpreted with caution. Second, as our study was conducted exclusively in a Korean cohort, its findings may not be directly generalizable to other, more ethnically diverse populations. The spectrum and frequency of pathogenic variants in cancer predisposition genes can vary significantly across different ancestries. Third, our study was not designed to assess downstream clinical utility. Therefore, we did not collect data on subsequent changes in patient management, genetic counseling, or cascade testing in at-risk family members, all of which are critical measures of the broader clinical impact of genetic testing. Finally, we did not include tumor genomic sequencing data, which prevented the analysis of somatic-germline correlations and an assessment of whether a “tumor-first” testing approach might have identified the pathogenic variants found in our cohort. Future studies with larger, multi-ethnic cohorts that incorporate both clinical utility outcomes and somatic tumor sequencing are necessary to validate and expand upon our findings.

## 5. Conclusions

This prospective study shows that germline genetic testing based exclusively on MPC status yields a pathogenic variant detection rate comparable to traditional guideline-based testing approaches. This finding suggests that the presence of MPC per se should be recognized as an independent indicator for hereditary cancer genetic testing, even in patients who do not fulfill cancer-specific guidelines. Adopting broader MPC-based testing strategies could significantly enhance the identification of hereditary cancer syndromes and improve early detection, preventive care, and clinical management. Larger-scale studies and population-specific guideline development are necessary to validate these findings and refine genetic testing recommendations.

## Figures and Tables

**Table 1:** Baseline characteristics and genetic testing results of multiple primary cancer patients for different testing criteria.

Characteristics	Guideline Group *n* = 29		Non-Guideline Group *n* = 33		*p*-Value
Median age at testing (range, years)	63 (39~84)		70 (49~84)		0.001
Median age at 1st cancer diagnosis (years)	53 (29~80)		61 (46~82)		0.002
Median age at 2nd cancer diagnosis (years)	59 (32~80)		67 (48~82)		0.006
Sex, *n* (%)					0.004
Male	7	24.1%	21	63.6%	
Female	22	75.9%	12	36.4%	
Number of primary cancers, *n* (%)					0.565
Two primary cancers	24	82.8%	30	90.9%	
Three or more primary cancers	5	17.2%	3	9.1%	
Family history of cancer (1st degree), *n* (%)					0.633
Yes	15	51.7%	14	42.4%	
No	14	48.3%	19	57.6%	
Germline results, *n* (%)					0.763
PV/LPV	2	6.9%	2	6.1%	
VUS	13	44.8%	12	36.4%	
ND	14	48.3%	19	57.6%	
Patients with family history (*n* = 29), *n* (%)					0.544
PV/LPV	2	13.3%	2	14.3%	
VUS	6	40.0%	3	21.4%	
ND	7	46.7%	9	64.3%	

Abbreviations: PV, pathogenic variant; LPV, likely pathogenic variant; VUS, variant of uncertain significance; ND, not detected.

**Table 2:** Detailed clinical and genetic information of multiple primary cancer patients with pathogenic germline variants.

Group	Sex	Age	Gene	Variant	Site of Primary Malignancies (Age at Diagnosis)	Family History
Guideline group	F	72	*CHEK2* (NM_007194.4)	c.846 + 1G > T	Breast (61) Ovary (61)	sister—breast cancer
	F	56	*BRCA2* (NM_000059.3)	c.5576_5579del, p.(Ile1859Lysfs)	Breast (36) Pancreas (54)	mother—breast cancer
Non-Guideline group	F	79	*ATM* (NM_000051.4)	c.103C > T, p.(Arg35Ter)	Tongue (59) Breast (79)	brother—gastric cancer (70 s) brother—prostate cancer(early stage, 70 s)
	M	70	*TP53* (NM_000546.6)	17p13.1del	Stomach (53) Nasal cavity (54) NSCLC (60) Tongue (64)	son—lung cancer

## Data Availability

The data that support the findings of this study are available from the corresponding author upon reasonable request.

## Supplementary Materials

The following supporting information can be downloaded at: https://www.mdpi.com/article/10.3390/jcm14207310/s1, Table S1. Variant-level list for all germline findings detected in the cohort, including HGVS nomenclature, ClinVar accessions/classifications, and population allele frequencies; Table S2. Frequency and types of primary cancers in the study cohort and by genetic testing criteria groups; Table S3. Additional information of variants identified in the cohort with reported tumor associations, ClinVar annotations, and literature references.
