# Supplementary material for: Multiple Primary Cancers as an Independent Criterion for Germline Testing: Comparison with Guideline-Based Criteria
Source: J Clin Med. 2025 Oct 16;14(20):7310. doi: 10.3390/jcm14207310 (PMC12565061; doi:10.3390/jcm14207310)
Supplement: Supplementary file 1 [file jcm-14-07310-s001.zip › jcm-3796461-supplementary.pdf]

Multiple Primary Cancers as an Independent Criterion for Germline Testing: Comparison with Guideline-Based Criteria

Supplementary materials

Table S1. Variant-level list for all germline findings detected in the cohort, including HGVS nomenclature, ClinVar accessions/classifications, and population allele frequencies.

Group	Gene	Pathogenicity	Transcript	Nucleotide Change	Amino Acid Change	Zygosity	rs ID	AF_K*	AF_EAS*	AF_total*	Clinvar Accession	Clinvar Classification
GG	*MSH6*	VUS	NM_000179.3	c.3772C>G	p.(Gln1258Glu)	heterozygote	rs63750554	0.002096	0.0004893	0.00003981	VCV000237198.33	Conflicting classifications of pathogenicity<br>Uncertain significance(8); Benign(1)
GG	*CHEK2*	VUS	NM_007194.4	c.1357G>C	p.(Ala453Pro)	heterozygote	rs763395924	0.0007858	0.0001631	0.00001195	VCV000232008.36	Conflicting classifications of pathogenicity<br>Uncertain significance(11); Likely benign(1)
GG	*APC*	VUS	NM_000038.6	c.6754C>G	p.(Pro2252Ala)	heterozygote	rs587778035	0.0005249	0.0001088	0.00000797	VCV000133520.14	Conflicting classifications of pathogenicity<br>Uncertain significance(2); Benign(1); Likely benign(1)
GG	*NBN*	VUS	NM_002485.5	c.511A>G	p.(Ile171Val)	heterozygote	rs61754966	0.00681	0.001453	0.0015	VCV000006946.91	Conflicting classifications of pathogenicity<br>Uncertain significance(15); Benign(3); Likely benign(6)
GG	*MSH2*	VUS	NM_000251.3	c.1408G>A	p.(Val470Ile)	heterozygote	rs1391167729	0	0	0	VCV000455491.15	Uncertain significance
GG	*NBN*	VUS	NM_002485.5	c.505C>T	p.(Arg169Cys)	heterozygote	rs182756889	0.0005546	0.0006515	0.00007784	VCV000127873.40	Conflicting classifications of pathogenicity<br>Uncertain significance(11); Likely benign(1)
GG	*MUTYH*	VUS	NM_001128425.2	c.493G>A	p.(Ala165Thr)	heterozygote	rs201103359	0.0005238	0.000351	0.00004257	VCV000185959.32	Uncertain significance
GG	*PALB2*	VUS	NM_024675.4	c.2509G>A	p.(Glu837Lys)	heterozygote	rs587778587	0.004191	0.001103	0.00009932	VCV000128133.40	Conflicting classifications of pathogenicity<br>Uncertain significance(4); Likely benign(11)
GG	*MUTYH*	VUS	NM_001128425.2	c.1477G>A	p.(Val493Ile)	heterozygote	rs587782228	0	0	0.000004172	VCV000142092.17	Uncertain significance
GG	*PMS2*	VUS	NM_000535.7	c.1688_1689delinsAG	p.(Arg563Gln)	heterozygote	rs587780725	NA	NA	NA	VCV000135940.34	Conflicting classifications of pathogenicity<br>Uncertain significance(3); Likely benign(5)
GG	*CDK4*	VUS	NM_000075.4	c.763C>T	p.(Arg255Cys)	heterozygote	rs587778188	0.001048	0.0007017	0.00005319	VCV000133877.30	Conflicting classifications of pathogenicity<br>Uncertain significance(4); Benign(1); Likely benign(4)
GG	*NBN*	VUS	NM_002485.5	c.511A>G	p.(Ile171Val)	homozygote	rs61754966	0.00681	0.001453	0.0015	VCV000006946.91	Conflicting classifications of pathogenicity<br>Uncertain significance(15); Benign(3); Likely benign(6)
GG	*MRE11*	VUS	NM_005591.4	c.469A>G	p.(Met157Val)	heterozygote	rs147771140	0.001576	0.001956	0.000177	VCV000142476.30	Conflicting classifications of pathogenicity<br>Uncertain significance(3); Likely benign(3)
GG	*ATM*	VUS	NM_000051.4	c.2804C>G	p.(Thr935Arg)	heterozygote	rs3218708	0.001049	0.0002719	0.00001989	VCV000185083.38	Conflicting classifications of pathogenicity<br>Uncertain significance(5); Likely benign(2)
NGG	*ATM*	VUS	NM_000051.4	c.3575A>G	p.(Lys1192Arg)	homozygote	rs1555091451	NA	NA	NA	VCV000489535.17	Uncertain significance
NGG	*CHEK2*	VUS	NM_007194.4	c.1111C>T	p.(His371Tyr)	heterozygote	rs531398630	0.003929	0.00421	0.0004526	VCV000128044.70	Conflicting classifications of pathogenicity<br>Uncertain significance(13); Benign(1); Likely benign(3)
NGG	*ATM*	VUS	NM_000051.4	c.3575A>G	p.(Lys1192Arg)	homozygote	rs1555091451	NA	NA	NA	VCV000489535.17	Uncertain significance
NGG	*BRIP1*	VUS	NM_032043.3	c.2024A>C	p.(Glu675Ala)	heterozygote	rs1555601081	NA	NA	NA	VCV000481653.14	Uncertain significance

NGG	*CHEK2*	VUS	NM_007194.4	c.1111C>T	p.(His371Tyr)	heterozygote	rs531398630	0.003929	0.00421	0.0004526	VCV000128044.70	Conflicting classifications of pathogenicity<br>Uncertain significance(13); Benign(1); Likely benign(3)
NGG	*CDH1*	VUS	NM_004360.5	c.1478T>C	p.(Val493Ala)	heterozygote	rs786202407	0.0002619	0.0001087	0.000007953	VCV000185725.20	Uncertain significance
NGG	*BRIP1*	VUS	NM_032043.3	c.2554A>G	p.(Asn852Asp)	heterozygote	rs745782331	0.001048	0.0002175	0.00001591	VCV000232278.21	Uncertain significance
NGG	*MSH2*	VUS	NM_000251.3	c.1004C>T	p.(Thr335Ile)	heterozygote	rs63750602	0	0	0.000003977	VCV000090499.9	Uncertain significance
NGG	*MSH2*	VUS	NM_000251.3	c.1144C>T	p.(Arg382Cys)	heterozygote	rs752373431	0.0002621	0.00005012	0.00003182	VCV000232371.45	Conflicting classifications of pathogenicity<br>Uncertain significance(8); Likely benign(2)
NGG	*RAD50*	VUS	NM_005732.4	c.353T>C	p.(Ile118Thr)	heterozygote	rs200472836	0.004191	0.0009253	0.00006787	VCV000142082.20	Uncertain significance
NGG	*MUTYH*	VUS	NM_001128425.2	c.934-2A>G	p.(?)	heterozygote	rs77542170	0.01388	0.01539	0.001103	VCV000041766.87	Conflicting classifications of pathogenicity<br>Uncertain significance(7); Likely benign(7)
NGG	*BMPR1A*	VUS	NM_004329.3	c.563G>A	p.(Arg188His)	heterozygote	rs749780872	0	0.000435	0.00003581	VCV000219715.27	Conflicting classifications of pathogenicity<br>Uncertain significance(3); Likely benign(4)
NGG	*MUTYH*	VUS	NM_001128425.2	c.1072T>C	p.(Phe358Leu)	heterozygote	rs587781601	0.001312	0.0002732	0.0000201	VCV000141246.21	Uncertain significance
NGG	*CHEK2*	VUS	NM_007194.4	c.1111C>T	p.(His371Tyr)	heterozygote	rs531398630	0.003929	0.00421	0.0004526	VCV000128044.70	Conflicting classifications of pathogenicity<br>Uncertain significance(13); Benign(1); Likely benign(3)
NGG	*ATM*	VUS	NM_000051.4	c.2804C>G	p.(Thr935Arg)	heterozygote	rs3218708	0.001049	0.0002719	0.00001989	VCV000185083.38	Conflicting classifications of pathogenicity<br>Uncertain significance(5); Likely benign(2)
NGG	*MLH1*	VUS	NM_000249.4	c.2110G>C	p.(Val704Leu)	heterozygote	rs587781811	0.0002651	0.0000545	0.000003983	VCV000141517.18	Uncertain significance
NGG	*MLH1*	VUS	NM_000249.4	c.649C>T	p.(Arg217Cys)	heterozygote	rs4986984	0.008381	0.004411	0.0003398	VCV000090303.53	Conflicting classifications of pathogenicity<br>Uncertain significance(2); Benign(6); Likely benign(3)
NGG	*APC*	VUS	NM_000038.6	c.7150T>A	p.(Leu2384Ile)	heterozygote	rs755345693	0.001839	0.000381	0.00002797	VCV000428142.24	Conflicting classifications of pathogenicity<br>Uncertain significance(5); Benign(1); Likely benign(3)
NGG	*APC*	VUS	NM_000038.6	c.3314G>A	p.(Arg1105Gln)	heterozygote	rs548176472	0.0002623	0.0001089	0.00001198	VCV000188375.36	Conflicting classifications of pathogenicity<br>Uncertain significance(5); Likely benign(1)
GG	*CHEK2*	Pathogenic	NM_007194.4	c.846+1G>T	p.(?)	heterozygote	rs864622149	NA	NA	NA	VCV000530044.14	Pathogenic/Likely pathogenic
GG	*BRCA2*	Pathogenic	NM_000059.3	c.5576_5579del	p.((Ile1859Lysfs)	heterozygote	rs80359520	0.0002621	0.00005508	0.00001634	VCV000037975.96	Pathogenic
NGG	*TP53*	Pathogenic	NM_000546.6	c.-28-26_*75del (Exon 2-11)	p.(?)	heterozygote	NA	NA	NA	NA	NA	NA
NGG	*ATM*	Pathogenic	NM_000051.4	c.103C>T	p.(Arg35Ter)	heterozygote	rs55861249	0	0	0.0000199	VCV000003025.75	Pathogenic/Likely pathogenic

Abbreviations: GG, guideline group; NGG, Non-guideline group; VUS, variant of uncertain significance; AF_K, allele frequency of Korean population; AF_EAS, allele frequency of East Asian population; AF_total, allele frequency of global population; NA, Not available.

*Allele frequencies were obtained from gnomAD v2.1.1 (GRCh37).

Table S2. Frequency and types of primary cancers in the study cohort and by genetic testing criteria groups.

	All groups		Guideline Group		non-Guideline Group	
	n=132		n=63		n=69	
Breast cancer	22	16.7%	18	28.6%	4	5.8%
Thyroid cancer	15	11.4%	10	15.9%	5	7.2%
Lung cancer	14	10.6%	2	3.2%	12	17.4%
Head and Neck cancer	13	9.8%	1	1.6%	12	17.4%
Gastric cancer	12	9.1%	5	7.9%	7	10.1%
Colorectal cancer	10	7.6%	6	9.5%	4	5.8%
Urothelial cancer	9	6.8%	4	6.3%	5	7.2%
Pancreatic cancer	7	5.3%	7	11.1%	0	0.0%
Ovary cancer	6	4.5%	6	9.5%	0	0.0%
Biliary tract cancer	5	3.8%	0	0.0%	5	7.2%
Prostate cancer	4	3.0%	1	1.6%	3	4.3%
Esophageal cancer	3	2.3%	0	0.0%	3	4.3%
Hepatocellular carcinoma	3	2.3%	0	0.0%	3	4.3%
Renal cell carcinoma	2	1.5%	1	1.6%	1	1.4%
Sarcoma	2	1.5%	0	0.0%	2	2.9%
Cervix cancer	1	0.8%	0	0.0%	1	1.4%
Mesothelioma	1	0.8%	0	0.0%	1	1.4%
Melanoma	1	0.8%	0	0.0%	1	1.4%
Thymoma	1	0.8%	1	1.6%	0	0.0%
Neuroendocrine tumors	1	0.8%	1	1.6%	0	0.0%

Table S3. Additional information of variants identified in the cohort with reported tumor associations, ClinVar annotations, and literature references.

Gene	HGVS	Variant-specific condition reported in Clinvar	Typical tumor spectrum	OMIM phenotype	Associated syndrome	Reference source
CHEK2	NM_007194.4:c.846+1G>T	Familial cancer of breast; Hereditary cancer-predisposing syndrome; *CHEK2*-related cancer predisposition	Breast cancer, Prostate cancer	Tumor predisposition syndrome 4, breast/prostate/colorectal	*CHEK2*-Related Cancer Predisposition	PMID: 40440438, 33471991
BRCA2	NM_000059.3:c.5576_5579del	Breast-ovarian cancer, familial, susceptibility to, 2; Hereditary breast ovarian cancer syndrome; Familial cancer of breast	Breast cancer, Ovarian cancer	{Breast-ovarian cancer, familial, 2}	*BRCA2*-Associated Hereditary Breast and Ovarian Cancer	PMID: 20301425, 33471991
ATM	NM_000051.4:c.103C>T	Hereditary cancer-predisposing syndrome; Ataxia-telangiectasia syndrome	Breast cancer, Hematologic malignancies	Ataxia-telangiectasia, {Breast cancer, susceptibility to}	Ataxia-telangiectasia	PMID: 10217116, 20301790, 33471991
TP53	NM_000546.6:c.-28-26_*75del	NA	Adrenocortical carcinomas, Breast cancer, Central nervous system tumors, Osteosarcomas, Soft-tissue sarcomas	Li-Fraumeni syndrome	Li-Fraumeni syndrome	PMID: 20301488

Abbreviations: NA, Not available.
